# Fibrolipomatous hamartoma in the median nerve in the arm - an unusual location but with MR imaging characteristics: a case report

**DOI:** 10.1186/1749-7221-5-1

**Published:** 2010-01-12

**Authors:** Jessica Nilsson, Kristina Sandberg, Lars B Dahlin, Nina Vendel, Eva Balslev, Lone Larsen, Niels Søe Nielsen

**Affiliations:** 1 Hand Surgery, Department of Clinical Sciences in Malmö, Lund University, Malmö, Sweden; 2 Department of Anesthesiology, Intensive Care and Operations, Gentofte Hospital, Copenhagen, Denmark; 3 Department of Pathology, Herlev Hospital, Denmark; 4 Department of Radiology, Herlev Hospital, Denmark; 5 Department of Orthopaedics at Herlev Hospital, Division of Hand Surgery, Gentofte Hospital, Hellerup, Denmark

## Abstract

Fibrolipomatous hamartoma of the median nerve are usually located distally in the forearm and may have characteristic features on MR imaging. Here we report a patient with an extensive fibrolipomatous hamartoma at an unusual location proximally in the arm, where a preoperative MR imaging was pathognomonic and diagnosis was verified by an incisional biopsy. We suggest that MRI should be performed in cases with nerve dysfunction without an obvious cause after a thorough clinical examination.

## Background

The two most common nerve tumours in the upper extremity are Schwannoma and neurofibroma [1,2]. More rare is a fibrolipomatous hamartoma, a benign, slow-growing mass, which is usually located in the median nerve distally in the forearm [3-7] and in its digital branches [1,4,5]. With MR imaging it is not always possible to make a diagnosis of a nerve tumour [2], but the MR imaging characteristics of fibrolipomatous hamartoma are considered to be pathognomonic [3]. In coronal plane, the nerve tumour is characterised by serpiginous structures [4,6] (thickened nerve fascicles), which are surrounded by fat (high signal intensity on T1-weighted images, low signal intensity on fat-suppressed T2-weighted images) [3]. In most of the cases the fat is distributed between the nerve fascicles making the nerve tumour looking like a coaxial cable in the axial plane [3-5,8,9]. Even if the nerve tumour has a characteristic feature on MRI the suspicion of a nerve tumour is not always obvious for the clinician. Here we report a case with obscure clinical symptoms and signs of isolated median nerve dysfunction, where the MR imaging showed the characteristic features of a fibrolipomatous hamartoma in the arm and the diagnosis was verified by an incisional biopsy.

## Case presentation

A 57 year right-handed secretary was referred to our hospital July 2008. She described symptoms since November 2002 with paresthesia in the right index, long and ring (half of it) fingers. Furthermore, she told about fibrillations in the interphalangeal joint of the right thumb and the index finger, loss of FPL and FDP function to the index finger followed by atrophy of the thenar muscles a year later. She was operated with carpal tunnel release at another hospital April 2007 due to a suspicion of a carpal tunnel syndrome, but no neurography or electromyography (EMG) was performed. In addition, she was operated with division of the A1 pulley on the right thumb due to a suspicion of a right-sided trigger thumb, but with no improvement. In February 2008, after the carpal tunnel release, neurography and EMG were performed. These investigations showed a severe loss of nerve fibres, but with remaining nerve fibers, in the right median nerve. Electrophysiologically, signs of reinnervation were seen, but no nerve compression lesion was found.

**Figure 1:**
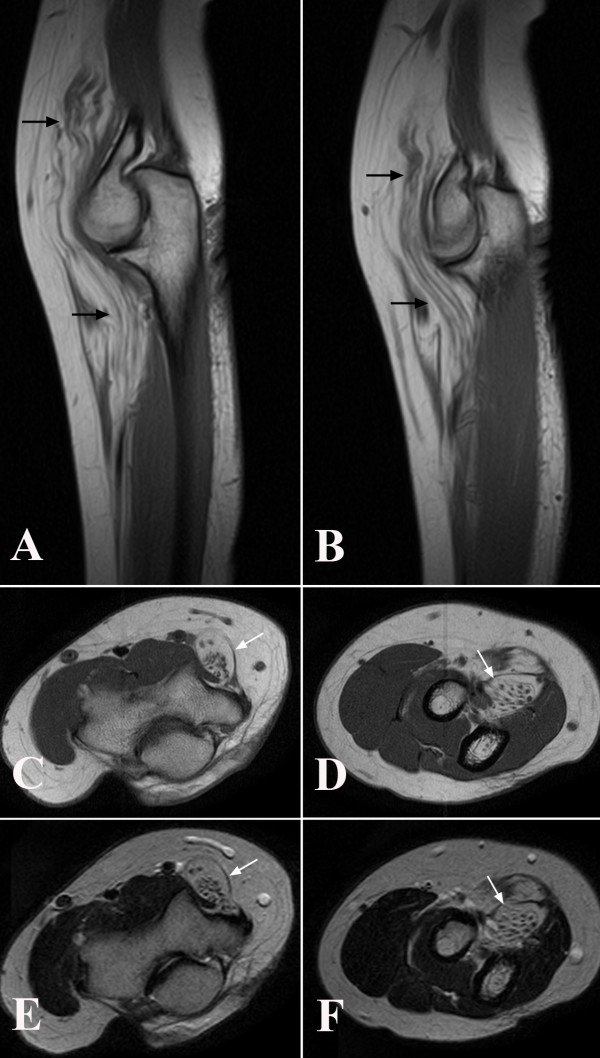
**Magnetic resonance investigation (MRI) of the patient showing specific characteristics of a fibrolipomatous hamartoma (arrows) in sagittal sections (T1 weighted in A and B) and in axial sections (T1-weighted in C and D; T2-weighted in E and F) at the elbow region**.

**Figure 2:**
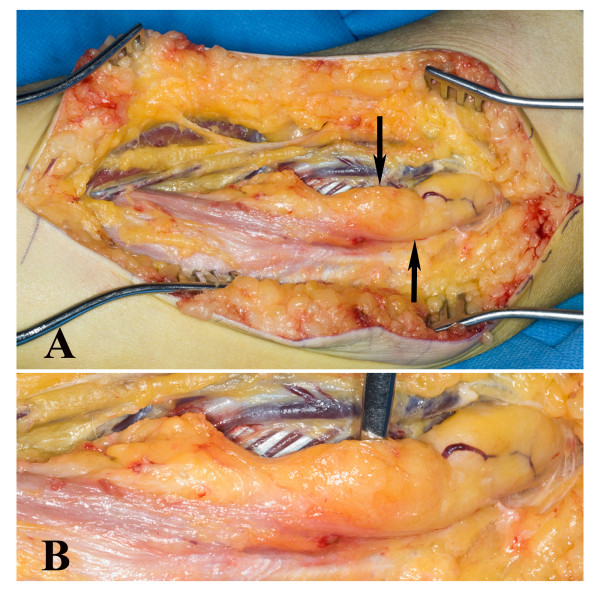
**At exploration of the median nerve at the elbow region a thickened nerve (arrows in A) was shown where the incisional biopsy showed a fibrolipomatous hamartoma (see Figure 3)**. Close up of the condition in B.

**Figure 3:**
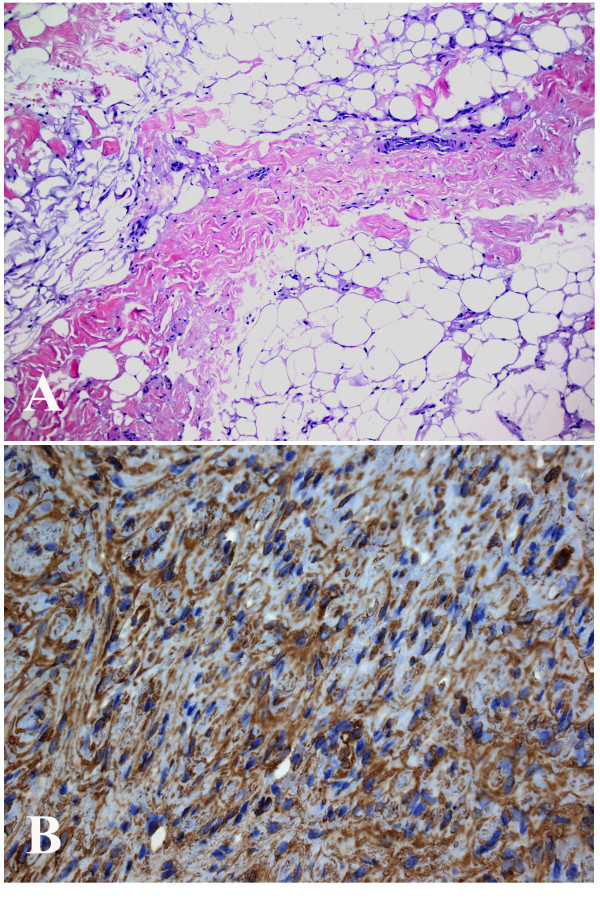
**Microscopical pictures of sections from the incisional biopsy showing (A) fibrolipomatous tissue with few atrophic peripheral nerve fascicles and fibrotic tissue (HE; × 10) and (B) atrophic and fibrotic nerve fibers stained with S-100 immunohistochemical staining (S-100; × 20)**.

At the clinical examination in July 2008 at our hospital she had atrophy of the thenar muscles and clear signs of affection of the anterior interosseous nerve with impaired function of the FDP to the index finger and FPL and decreased grip strength. She described a slight pain at palpation in the middle part of the forearm along the median nerve. She had paraesthesia located only in the long finger. The circumference of the right arm was 1.5 cm shorter than the left forearm indicating atrophy of some of the forearm muscles. She had no other clinical or neurographical signs of motor or sensory dysfunction. There was no obvious cause of the longstanding, severe nerve fibre loss in the median nerve. An MRI was performed of the right median nerve at axilla level and distally. The MRI showed a median nerve with serpiginous appearance. Single nerve fascicles in a well defined tissue mass containing fat was found (Figure 1). The condition extended from the proximal humerus to the wrist and thereafter the median nerve had a normal appearance. The diameter of the tumour was 1.7-2.5 cm. Due to the typical MRI changes (Figure 1) the diagnosis was suggested as a fibrolipomatous hamartoma of the median nerve. An incisional biopsy after exploration of the median nerve was done in February 2009 under microscopical dissection. The median nerve had a diameter up to 2 cm and there were no adhesions to the surrounding tissue. The macroscopical appearance of the nerve tumour indicated a fibrolipomatous hamartoma (Figure 2) and five incisional biopsies were taken from representative areas. Neurolysis of the median nerve was done from the middle part of the arm down to the middle part of the forearm. Microscopy showed a fibrolipomatous tissue that surrounded and splayed apart the peripheral nervous tissue, which was also fibrotic and atrophic (Figure 3). The postoperative follow-up was uneventful. She returned to her original profession. She still has dysfunction of the FPL, and thenar atrophy although she can do an opposition of the thumb. She felt some improvement after the exploration and neurolysis of the nerve around the elbow. A tendon transfer procedure may be considered in the future. She is followed regularly with particularly clinical examinations.

## Discussion

Of all tumours in the upper extremity, 2% are nerve tumours [10]. Schwannoma is probably the most frequent one with a known incidence of less than 1/100000 inhabitants and year in Sweden [1]. Usually, the diagnosis of a nerve tumour has to be based on microscopical findings since MRI is not sufficient for a precise diagnosis [2]. In contrast, a fibrolipomatous hamartoma, which is even more rare, has very distinct characteristics in MRI with serpiginous hypotense structures representing thickened fascicles which are surrounded by evenly distributed fat [4] (high T1-weighted signal intensity and low fat-suppressed T2-weighted signal intensity) [3,4,6]. Our case showed such MRI characteristics and, furthermore, the diagnosis was confirmed by microscopy. We did not perform any ultrasound examination of the patient due to the lack of palpable tumour before exploration. However, such an investigation may be considered when there is a suspicion of a nerve tumour, although MRI is frequently used [2].

The origin of fibrolipomatous hamartoma is still obscure and is mainly affecting and found in young persons [4-7]. That might indicate a congenital aetiology [7], although a few cases have been reported in older people as seen here. Another theory is that the lesion can be caused by trauma [3-5]. There are debates of the relationship between fibrolipomatous hamartoma and macrodactyli [3-5,7]. However, our patient had no signs of this clinical presentation.

Our patient had a long history with motor disturbances, such as thenar atrophy and loss of FPL function. Most cases present with a longstanding painless mass. Nerve compression of the affected nerve with paresthesia, motor deficit and pain are known late symptoms [4,5,7,11-14]. It is suggested that it may become symptomatic only in the median nerve due to encroachment by the flexor retinaculum; thus causing carpal tunnel syndrome [4,5]. However, in our case the tumour was located more proximally extending from the upper arm almost down to wrist level; thus, presenting a more proximal located fibrolipomatous hamartoma than previously described. A hypothesis could be that you rarely find these fibrolipomatous hamartoma more proximal because of spatial relations.

Only a limited number of cases of fibrolipomatous hamartoma are reported in the literature showing the uncertainty of the optimal treatment suggestion and that treatment should be guided by the severity of symptoms [5,15]. Our case was treated only by exploration and release of potential narrowing structures, particularly around the elbow, which improved her symptoms, but excision of the tumour is not recommendable [3-7,16,17]. Due to the extensive fatty infiltration of the nerve fascicles, surgical excision may cause catastrophic motor and sensory deficits. We performed five incisional biopsies from different locations to be sure of adequate material for the neuropathological examination. When an incisional biopsy is gently performed it is our experience that no further dysfunction is added to the patient. We will follow our patient regarding any progression of the tumour and further consider additional treatment, such as tendon transfers, for the impaired function of FPL and FDP to the index finger. However, the long-term results are obscure of fibrolipomatous hamartoma. Meticulous information to the patient and a regular follow-up are recommended.

The present case emphasizes the need for a thorough history from the patient and a careful and meticulous clinical examination of cases with symptoms from the peripheral nervous system. For example, a nerve tumour can be the cause of symptoms as the present case. A MRI may reveal a nerve structure with a coaxial-cable-like appearance; thus with a high suspicion of the diagnosis of fibrolipomatous hamartoma.

## Conclusions

Although history of patients with symptoms from the peripheral nervous system as well as a meticulous clinical examination is recommended, an MRI is an additional tool to reveal a fibrolipomatous hamartoma at an unusual location.

## Consent

Written informed consent was obtained from the patient for publication of this case report and any accompanying images. A copy of the written consent is available for review by the Editor-in-Chief of this journal.

## Competing interests

The authors declare that they have no competing interests.

## Authors' contributions

The medical students JN and KS and senior author LD have done literature review and written the draft of the manuscript. The patient was operated by NSN (senior author) and NV (nurse; literature review). MRI was performed by LL and the microscopical examination by EB. All authors have contributed in different important ways to the present manuscript.

All authors have read and approved the final manuscript.
